# ^18^F-FDG and ^18^F-FAMT PET-derived metabolic parameters predict outcome of oral squamous cell carcinoma

**DOI:** 10.1007/s11282-019-00377-2

**Published:** 2019-02-18

**Authors:** Mai Kim, Tetsuya Higuchi, Takahito Nakajima, Putri Andriana, Hiromi Hirasawa, Azusa Tokue, Jun Kurihara, Satoshi Yokoo, Yoshito Tsushima

**Affiliations:** 1 Department of Oral and Maxillofacial Surgery and Plastic Surgery, Gunma University Graduate School of Medicine, 3-39-22 Showa-machi, Maebashi, Gunma 371-8511 Japan; 2 Department of Diagnostic Radiology and Nuclear Medicine, Gunma University Graduate School of Medicine, 3-39-22 Showa-machi, Maebashi, Gunma 371-8511 Japan

**Keywords:** Oral squamous cell carcinoma, Positron emission tomography (PET), Volumetric PET parameter, Prognosis, ^18^F-FDG, ^18^F-FAMT

## Abstract

**Objectives:**

L-3-[^18^F]-Fluoro-α-methyl tyrosine (FAMT), an amino acid positron emission tomography (PET) tracer, complements [^18^F]-fluorodeoxyglucose (FDG) in the diagnosis of malignancies. We compared the predictive ability of FAMT PET versus FDG PET regarding metastatic oral squamous cell carcinoma (OSCC) outcomes for distant metastasis, including lymph node metastasis, and identified the relevant metabolic parameters for each.

**Methods:**

We enrolled 160 patients with OSCC who underwent PET/computed tomography using FDG and FAMT before treatment. Outcomes were assessed using clinicopathological characteristics such as the standardized uptake value (SUV_max_, SUV_peak_), metabolic tumor volume (MTV), and total lesion glycolysis or total lesion retention. Univariate and multivariate Cox proportional hazards models were used to identify the independent predictors of disease-free survival (DFS) and overall survival (OS) during an average follow-up time of 1401.7 and 1646.0 days, respectively. Areas under the receiver operating characteristic curves were analyzed for the accuracy and predictive value of imaging parameters.

**Results:**

Clinical parameters (excluding age) and PET metabolic parameters were significantly associated with OS. Multivariate analysis showed that an infiltrative growth pattern [*p* = 0.034, hazard ratio (HR) = 2.30], and the FDG-measured SUV_peak_ (*p* = 0.045, HR = 2.45) were independent risk factors for DFS and that lymph node metastasis (*p* = 0.03, HR = 2.57) and the FAMT-measured MTV (*p* = 0.004, HR = 3.65) were independent risk factors for OS.

**Conclusions:**

In patients with OSCC, FDG PET predicted DFS, whereas FAMT predicted OS. The two PET tracers, combined with clinical parameters, provide complementary, outcome-related diagnostic information in OSCC.

## Introduction

Lip and oral cavity cancers have an incidence of 1.2% and represent 1.0% of all-cancer mortality. They were responsible for almost 3,945 deaths among the 409,399 cancer-related deaths in Japan in 2018 [1]. Oral squamous cell carcinoma (OSCC) represents approximately 30% of head and neck cancers and is the most frequent type of such cancers [2]. The use of prognostic parameters such as clinicopathological factors, anatomical location, and TNM stage in such cases still fails to identify those patients in need of intensive versus standard therapy prior to treatment [3].

[^18^F]-Fluorodeoxyglucose (FDG) is a radiologic analog of glucose through which positron emission tomography (PET) enables the visualization of glucose metabolism; it is useful as a marker of tumor metabolic activity in terms of cell viability and proliferation [4, 5]. Analyses of the metabolic tumor volume (MTV) and total lesion glycolysis (TLG) by ^18^FDG PET have been used to investigate the clinical and prognostic ability of the technique, as indicated by parameters such as survival and occult metastasis, in patients with OSCC [6].

FDG is the most widely used PET radiotracer for the diagnosis, characterization, and staging of malignant oral cancer because of its high sensitivity [7]. Nevertheless, the tracer lacks specificity because of its inability to identify the tumor as malignant or benign; FDG accumulates in both benign tumors and inflammatory lesions, causing false positives to occur [8]. Accurate assessment of local involvement is important to minimize the extent of surgery, given that surgical treatment usually involves the removal of oral cavity organs or mandibular segmentectomy in patients with oral cancers. Surgery is also hampered by the complexities of the head/neck anatomy, which increase the need for a precise diagnosis of tumor invasion [9]. Therefore, new PET radiotracers with higher specificity are needed for the accurate evaluation of tumor size.

L-[3-^18^F]-α-Methyl tyrosine (FAMT) is an amino acid tracer that has been developed for PET imaging and that shows better specificity for cancer diagnosis than FDG PET [10]. FAMT is incorporated into cancer cells by L-type amino acid transporter 1 (LAT1), which is only overexpressed in malignant tumors. This overexpression is triggered by cell proliferation and angiogenesis in various human cancers, including brain, lung, prostatic, breast, pancreatic, gastric, urinary tract, and esophageal cancers [11, 12]. Low expression of LAT1 has been reported in adenocarcinoma (29% in pulmonary adenocarcinoma [13], 22% in prostate cancer [14], 43% in breast cancer [15], 52% in pancreatic cancer [16], and 43% in gastric cancer [17]). Conversely, this transporter is highly expressed in squamous cell carcinoma (91% in pulmonary squamous cell carcinoma [13] and 50% in oral cancer [11]). We have reported that the specificity of FAMT PET for malignant tumors is higher than that of FDG PET and that tumor delineation by FAMT PET is also superior to that by FDG PET [11]. However, the ability of FAMT PET to predict disease-free survival (DFS) or overall survival (OS) outcomes in patients with OSCC is unknown. Therefore, this study was designed to compare the utility of FAMT PET-derived and FDG PET-derived metabolic parameters in predicting clinical outcomes in patients with OSCC.

## Materials and methods

### Study population

We retrospectively evaluated 160 patients who underwent FDG PET and FAMT PET with computed tomography (CT) before treatment at our institution from April 2008 to June 2015; the patient group comprised 96 men and 64 women with a median age of 69.2 years (range 27–93 years). The examined clinical factors were adjusted for age, sex, and the location of the tumor. OSCC was diagnosed based on pathological findings and was confirmed in all patients by a pathologist. The TNM stage was classified using the UICC 2009 TNM cancer staging system (ver. 7) during the data collection. The infiltrative growth pattern (INF) was used as a measured variable in the univariate analysis, and each tumor was classified as INF-a (extensive growth of tumor nests with a well-defined border from surrounding tissue), INF-b (intermediate growth pattern between INF-a and -c), or INF-c (infiltrative growth of tumor nests with an ill-defined border from surrounding tissue) [18]. The data obtained from the medical records were clinical variables, treatment, and follow-up events (lymph node metastasis or recurrence). Informed consent was obtained from all patients for involvement in this study. This study was approved by the institutional review board (IRB number: 2017 − 254).

### PET imaging and analysis

PET volumetric parameters were computed from attenuation-corrected PET data using a syngo.via device (SIEMENS Healthcare, Erlangen, Germany) and oncology software package (SIEMENS Healthcare). ^18^F-FDG or ^18^F-FAMT was intravenously administered to the patients at a dose of 5.0 MBq/kg (median dose: ^18^F-FDG, 276.6 MBq and ^18^F-FAMT, 255.5 MBq) after they had fasted for at least 6 h. PET imaging was performed 68.8 ± 16.5 and 67.6 ± 13.7 min after administering ^18^F-FDG and ^18^F-FAMT, respectively. PET volumetric parameters were calculated using ^18^F-FDG and ^18^F-FAMT thresholds of 2.5 and 1.4, respectively. These cut-off values were in accordance with those in our previous study, suggesting that the cut-off standardized uptake value (SUV) for patients with OSCC required an exclusive threshold [19]. This cut-off value was then used to calculate the PET parameters, which were derived by computerized-assisted reporting via threshold automated segmentation, to define volumes and then automatically calculate the MTV and average SUV. The resulting SUV (SUV_max_, SUV_peak_) and MTV were used to calculate the TLG for FDG and total lesion retention (TLR) for FAMT using the formula MTV × SUV _mean_ in both cases [20].

### Statistical analysis

Receiver operating characteristic curves were used for each metabolic tumor parameter. Univariate analysis was used to identify clinical, pathological, and PET volumetric factors that predict DFS and OS according to the Kaplan–Meier method and log-rank tests. Multivariate Cox proportional hazards model analysis was performed to determine significant odds ratios for DFS and OS. Time periods were calculated according to the Japan Clinical Cancer Research Organization guidelines. Statistical analyses were performed using SPSS software ver. 23 (IBM Corp., Armonk, NY, USA). In all tests, *p* < 0.05 was considered significant.

## Results

### Patient population

The demographic characteristics of the patients are shown in Table 1. The measured variables were age and sex, primary tumor site [most common was tongue (*n* = 60), followed by mandible (*n* = 49)], histological differentiation (mild and moderate, *n* = 144; severe, *n* = 16), INF and pathological TNM stage, and the treatment provided to patients.

**Table 1:** Tumor characteristics in the study population

Variables	All patients (*n* = 160)
Age, years	69.2 ± 12.8 (27–93)
Sex	
Male	96 (60.0)
Female	64 (40.0)
Site of primary tumor	
Tongue	60 (37.5)
Maxilla	26 (16.2)
Floor of mouth	13 (10.0)
Lip	3 (1.9)
Mandible	49 (30.6)
Buccal mucosa	9 (5.6)
Histological differentiation	
Mild	93 (58.1)
Moderate	51 (31.9)
Severe	16 (10.0)
Infiltrative growth (INF)	
INFa	22 (13.8)
INFb	72 (45.0)
INFc	66 (41.3)
Pathologic TNM stage	
T1/T2/T3/T4	34 (21.3)/64 (40.0)/16 (10.0)/46 (28.8)
N0/N1/N2	111 (69.4)/19 (11.9)/30 (18.7)
M0/M1	158 (98.8)/2 (1.3)
Stage I/II/III/IV	27 (16.9)/51 (31.9)/24 (15.0)/58 (36.1)
Treatment	
Surgery	140 (87.5)
Radiotherapy/chemoradiotherapy only	20 (12.5)

Data are presented as mean ± standard deviation (range) or *n* (%)

### Univariate and multivariate analyses

The univariate analysis results are shown in Table 2. Each parameter was evaluated in relation to DFS and OS for an average follow-up time of 1401.7 and 1646.0 days, respectively. The factors examined in the univariate analysis were age, sex, histological differentiation, pathological parameters (INF, tumor size, regional lymph node metastasis, and presence of distant metastasis), stage, and PET-derived parameters for FDG and FAMT (SUV_max_, SUV_peak_, MTV, and TLG/TLR). All of the tested parameters were significantly associated with OS regardless of age (*p* < 0.05).

**Table 2:** Univariate analysis of clinical, pathological, and PET parameters in relation to DFS and OS

Parameters	All patients (*n* = 160)	*p* value	
		DFS	OS
Age		0.762	0.174
≤75 years	95		
>75 years	65		
Sex		0.621	0.008*
Male	96		
Female	64		
Histological differentiation	0.001*	0.03*	
Well/moderately	144		
Poorly	16		
Infiltrative growth (INF)		0.003*	0.04*
INF-a/b	94		
INF-c	66		
Primary tumor		0.032*	0.001*
T1/T2/T3	114		
T4	46		
Lymph node metastasis		0.002*	0.001*
N0	111		
N1/N2a-c/N3	49		
Distant metastasis		0.235	0.003*
M0	158		
M1	2		
Stage		0.032*	0.003*
I/II/III	102		
IV	58		
FDG PET parameters			
SUV_max_ (g/ml)		0.010*	0.005*
≤8.25 (confirmed event)	76		
>8.25 (confirmed event)	84		
SUV_peak_ (g/ml)		0.005*	0.003*
≤4.81 (confirmed event)	62		
>4.81 (confirmed event)	98		
MTV (g/ml)		0.285	0.001*
≤8.51 (confirmed event)	80		
>8.51 (confirmed event)	80		
TLG (bw × cm^3^)		0.127	0.001*
≤29.8 (confirmed event)	77		
>29.8 (confirmed event)	83		
FAMT PET parameters			
SUV_max_ (g/ml)		0.082	0.001*
≤2.8 (confirmed event)	75		
>2.8 (confirmed event)	85		
SUV_peak_ (g/ml)		0.094	0.0007*
≤2.3 (confirmed event)	85		
>2.3 (confirmed event)	75		
MTV (g/ml)		0.014*	0.00001*
≤6.2 (confirmed event)	94		
>6.2 (confirmed event)	66		
TLR (bw × cm^3^)		0.040*	0.0002*
≤8.2 (confirmed event)	82		
>8.2 (confirmed event)	78		

*Cox proportional hazards model, *p* < 0.05 is considered significant

*DFS* disease-free survival, *OS* overall survival, *FDG PET*, [^18^F]-fluorodeoxyglucose positron emission tomography, *FAMT PET* L-3-[^18^F]-fluoro-α-methyl tyrosine positron emission tomography, *SUV* standardized uptake value, *MTV* metabolic tumor volume, *TLG* total lesion glycolysis, *TLR* total lesion retention

The FDG PET parameters MTV (g/mL) (≤ 8.51, *n* = 80; >8.51, *n* = 80) and TLG (bw × cm^3^) (≤ 29.8, *n* = 77; >29.8, *n* = 83) were not significantly associated with DFS, whereas SUV_max_ and SUV_peak_ were significantly associated with DFS (*p* = 0.010 and 0.005, respectively). The FAMT PET parameters SUV_max_ (g/mL) (≤ 2.8, *n* = 75; >2.8, *n* = 85) and SUV_peak_ (g/mL) (≤ 2.3, *n* = 85; >2.3, *n* = 75) were not significantly associated with DFS; however, MTV and TLR were significantly associated with DFS (*p* = 0.014 and 0.040, respectively). Of the pathological parameters, histological differentiation, INF, TNM stage, and overall stage were significantly associated with DFS (*p* < 0.05).

The multivariate analysis incorporated INF, regional lymph node metastasis, SUV_peak_ on FDG PET, and MTV on FAMT PET (Table 3). The results indicated that INF [hazard ratio (HR) = 2.30, *p* = 0.034] and SUV_peak_ (HR = 2.45, *p* = 0.045) were significantly associated with DFS and that lymph node metastasis (HR = 2.57, *p* = 0.03) and MTV (HR = 3.65, *p* = 0.004) were significant and independent risk factors for OS.

**Table 3:** Multivariate analysis of clinical, pathological, and PET parameters in relation to DFS and OS

Variables	DFS		OS	
	HR (95% CI)	*p* value	HR (95% CI)	*p* value
INF	2.30 (1.06–4.95)	0.034*	-	-
Lymph node metastasis	-	-	2.57 (1.09–6.04)	0.03*
FDG SUV_peak_	2.45 (1.02–5.89)	0.045*	-	-
FAMT MTV	-	-	3.65 (1.50–8.85)	0.004*

*PET* positron emission tomography, *DFS* disease-free survival; *OS* overall survival, *HR* hazard ratio, *CI* confidence interval, *INF* infiltrative growth, *FDG* [^18^F]-fluorodeoxyglucose, *SUV* standardized uptake value, *FAMT* L-3-[^18^F]-fluoro-α-methyl tyrosine, *MTV* metabolic tumor volume

### Representative cases

**Fig. 1:**
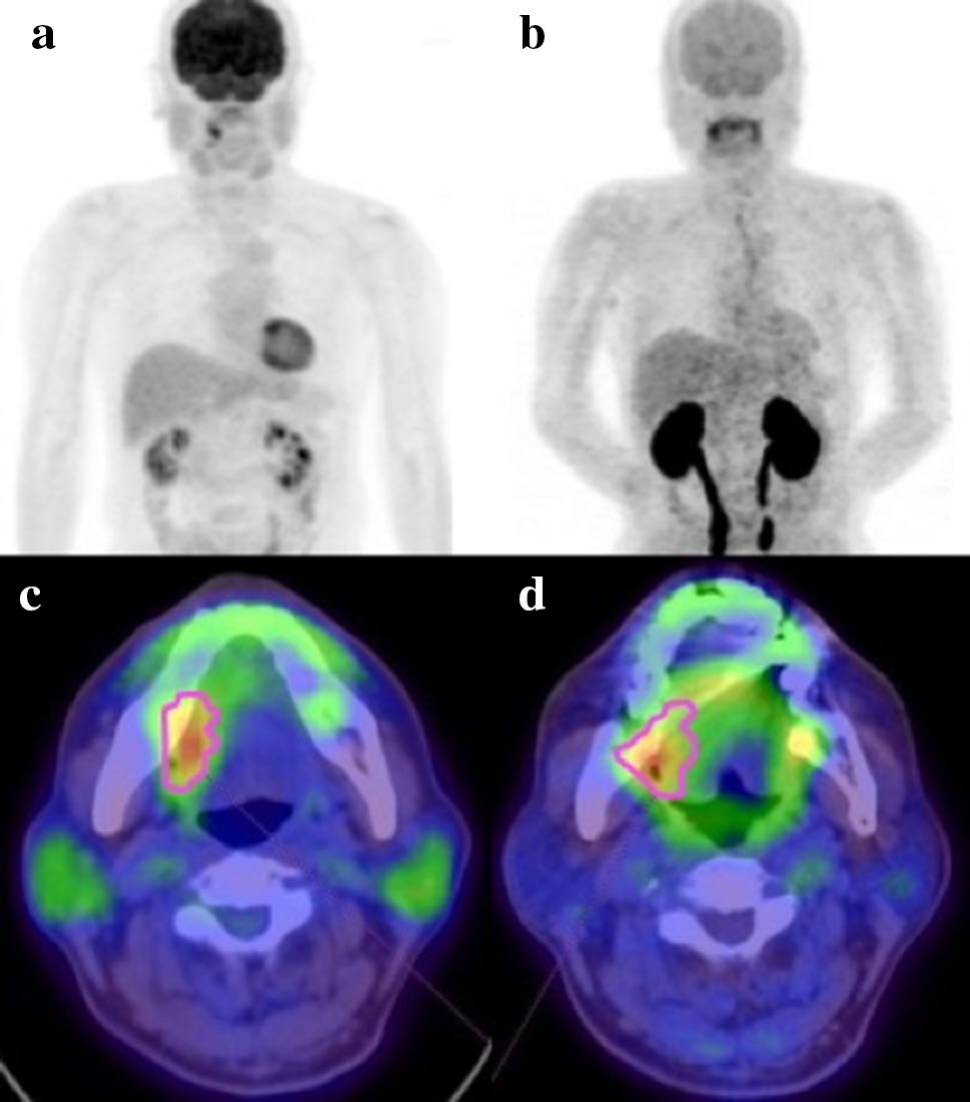
Maximum intensity projection imaging of a patient with a good outcome using **a**^18^F-FDG and **b**^18^F-FAMT and primary PET/CT imaging using **c**^18^F-FDG and **d**^18^F-FAMT

**Fig. 2:**
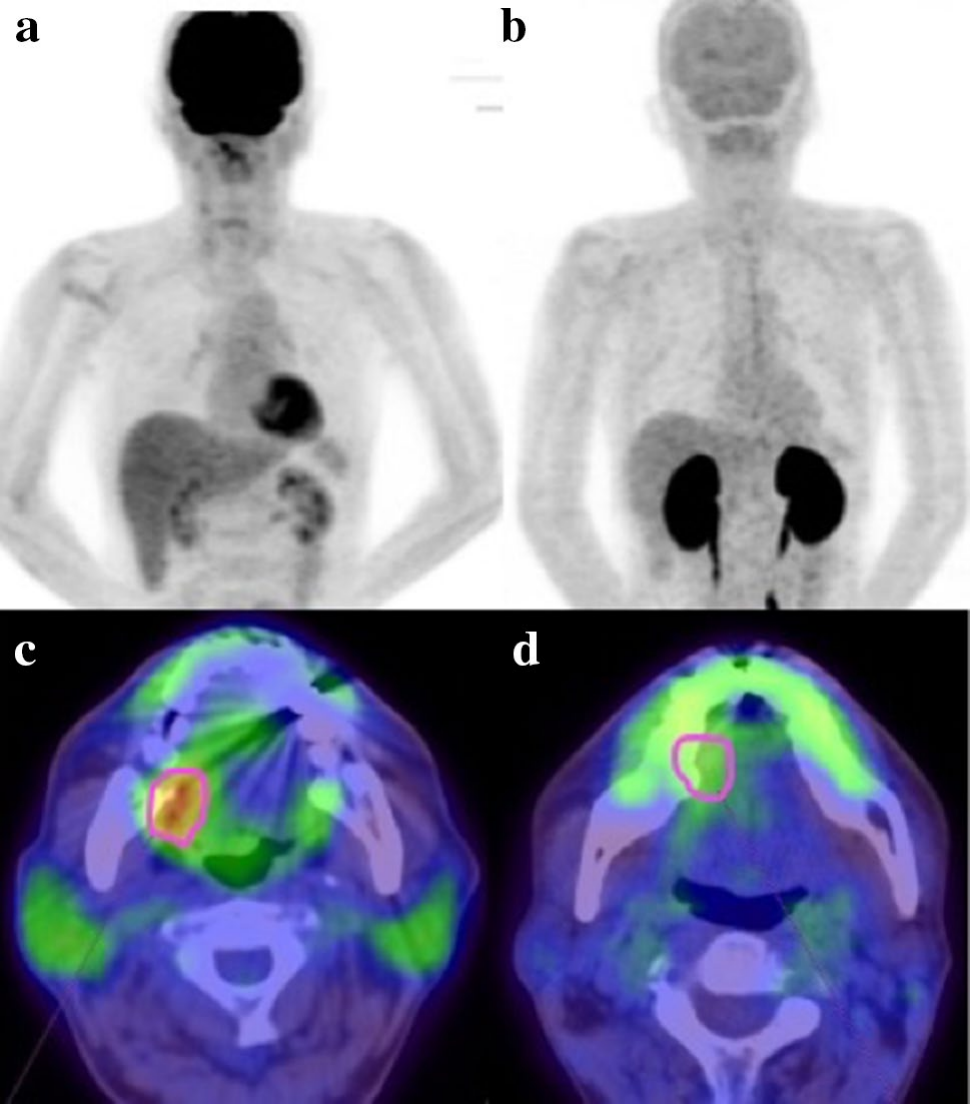
Maximum intensity projection imaging of a patient with a poor outcome using **a**^18^F-FDG, **b**^18^F-FAMT, and primary PET/CT imaging by **c**^18^F-FDG and **d**^18^F-FAMT

Figures 1 and 2 show representative cases in this study. Figure 1 shows a 51-year-old man with right tongue cancer (T4aN0M0, Stage IVA). ^18^F-FDG PET/CT scans showed intense accumulation of ^18^F-FDG (PET parameters: SUV_max_, 4.2 g/ml; SUV_peak_, 3.3 g/ml; MTV, 3.4 g/ml; TLG, 10.6 bw × cm^3^) (Fig. 1c); however, ^18^F-FAMT scans did not show intense accumulation (PET parameters: SUV_max_, 1.7 g/ml; SUV_peak_, 1.5 g/ml; MTV, 1.2 g/ml; TLR, 1.8 bw × cm^3^) in this patient (Fig. 1d). The patient outcomes were censored. Figure 2 shows a 63-year-old man with right tongue cancer (T2N2bM0, Stage IVA). ^18^F-FDG PET/CT scans showed intense accumulation of ^18^F-FDG (PET parameters: SUV_max_, 6.4 g/ml; SUV_peak_, 4.4 g/ml; MTV, 5.0 g/ml; TLG, 18.2 bw × cm^3^) (Fig. 2c), and ^18^F-FAMT scans also showed intense accumulation (PET parameters: SUV_max_, 2.3 g/ml; SUV_peak_, 1.8 g/ml; MTV, 6.1 g/ml; TLG, 10.5 bw × cm^3^) in this patient (Fig. 2d). The patient outcome was local recurrence (160 days postoperatively).

## Discussion

We found that FAMT uptake calculated in terms of MTV and lymph node metastasis were independent predictive factors for OS, while SUV_peak_ from FDG PET was not prognostic for OS. These results suggest that in patients with OSCC, FDG PET-derived SUV_peak_ is useful for predicting the short-term response because of the high sensitivity of the FDG radiotracer relative to FAMT, whereas FAMT PET is potentially clinically useful for predicting longer-term outcomes (e.g., survival) because of its relatively higher specificity.

FDG has been the gold standard for PET imaging of malignancies. However, detection of lesions with FDG depends on the glycolytic activity of inflammatory tissues that can potentially obscure tumors and increase false positives [18]. Insufficient standardization and reproducibility in determining the therapeutic response may also hamper the use of FDG as a prognostic marker [21]. We previously reported that OSCC lesions were overestimated when using FDG PET volumetric parameters because of localized inflammation [19]. FAMT was developed specifically to solve the issue of false positives in FDG PET [8, 12, 22, 23]. This is the first study to evaluate FAMT PET and its volumetric parameters as predictors of outcomes in OSCC. Volumetric parameters appear to be useful as prognostic markers in patients with OSCC given that parameters such as MTV and TLG reflect the whole tumor burden compared with SUV_max_. Volumetric parameters can be used during radiation or chemotherapy to directly visualize the metabolic reaction of the malignancy [24]. Eventually, such parameters might be assessed to derive whole tumor information, including one-point pixel information, which is considered to be a more reliable predictor of DFS and OS than is SUV_max_.

Pathological invasion is considered an independent prognostic factor in patients with OSCC. In clinical practice, the clinical and histopathological parameters derived from specimen biopsy and resection are the most common factors in deciding on a treatment strategy and determining the prognosis [25]. Lymph node metastasis and OS in patients with OSCC can also be assessed pathologically.

We hypothesize that FDG will prove to be better at defining localized lesions because of its high sensitivity and will be suitable for determination of DFS. In contrast, FAMT is likely to be more effective in evaluating the behavior of malignant tumors and would be more appropriate for determining OS. DFS itself is a better predictor of short-term therapeutic responses after initial therapy and can thus be a better strategy for predicting primary treatment effectiveness; it would also improve treatment guidance. OS, however, is a better predictor of long-term therapeutic responses. Hence, the combination of FDG PET and FAMT PET will provide superior predictive imaging results in the clinical setting.

This study had several limitations. First, this study was not directly focused on cell biomarkers (e.g., Ki-67 immunohistochemistry or LAT1 expression) that are closely associated with tumor cell proliferation, the grade of malignancy, and poor outcomes. Second, the semiquantitative evaluation in this study was dependent on the software used to derive the data. Thus, the thresholds used for PET volumetric parameter calculation were determined automatically, and accuracy would suffer if the thresholds were not correct. Therefore, a further study using a combination of clinical data and pathologic data is highly recommended. Finally, the images used automated tumor delineation based on fixed thresholds, which may have also reduced the accuracy.

FDG SUV_peak_ and FAMT MTV are significant predictors of DFS and OS, respectively, in patients with OSCC. Our data suggest that volumetric PET imaging parameters might be able to predict both short- and long-term outcomes and possibly do so as well as clinicopathologic predictors.
